# Medical student perceptions of research training on patient care during clerkship

**DOI:** 10.15694/mep.2020.000107.1

**Published:** 2020-05-21

**Authors:** Telisha Smith-Gorvie, Joyce Nyhof-Young, Jennifer Ng, Tony D'Urzo, Debra Katzman

**Affiliations:** 1 University of Toronto

**Keywords:** Curriculum development, Evidence-based medicine, Consumer of research, Evaluation, Undergraduate medical education, Patient care

## Abstract

This article was migrated. The article was marked as recommended.

Background

Health Science Research (HSR) is a pre-clerkship component of the University of Toronto (U of T) MD Program. Through online modules and tutorials, students learn to understand and apply research, and write an original research protocol. This study explored students’ perceptions on how HSR prepared them to identify, critically appraise and consume research during clerkship.

Methods

An online 12-item questionnaire surveyed U of T medical students (Class of 2018) who completed HSR in 2016. Basic descriptive statistics were performed; free text responses were analysed via descriptive thematic analysis.

Results

Twenty six percent (67/262) of students participated. Approximately half either agreed/strongly agreed that HSR helped them to critically appraise research articles (50.7%, 32/63) and assess applicability of results to patient care (50.8%, 32/63). Three themes emerged: i) desire for increased critical appraisal, ii) producing research less important than consuming research, iii) developing a greater appreciation of research during clerkship.

Conclusions

Students’ perceptions on HSR’s value during clerkship were modest; they desired greater focus on learning to be consumers of research. These results will refine HS, and our observations may be useful to other educators, as this type of intervention is not represented in existing literature.

## Introduction

### Background and introduction

Evidence-based medicine (EBM) represents the “conscientious, explicit, and judicious use of current best evidence” applied appropriately within the context of a clinician’s individual judgment and expertise ( Sackett, 1996). It also includes consideration of patient values and preferences ( Sackett, 1996; Alahdab *et al.*, 2012; Abu-Zaid *et al.*, 2013). EBM has become a necessary part of clinical practice for physicians ( Alonso-Coello *et al.*, 2009). Critical appraisal, in which research is systematically scrutinised with respect to a specific research question, its associated methodologies, findings and relevance to patient care, is rooted in the principles of evidence-based medicine (CEBM, 2019).

Some evidence suggests that teaching of research methods and EBM skills (including critical appraisal) should begin early in the undergraduate medical education (UME) curriculum ( Srinivasan *et al.*, 2002; Liabsuetrakul *et al.*, 2009; Abu Zaid, 2013; Black *et al.*, 2013). Benefits of such early exposures include the development of critical thinking skills, earlier appreciation of the relationship between research, clinical practice and EBM, improved student attitudes towards research, and increased student motivation to perform research ( Siemens *et al.*, 2010; Nazha *et al.*, 2015; Feigerlova *et al.*, 2016). Globally, there has been enhanced integration of EBM skills and research methods teaching into UME programs ( Chang and Ramnanan, 2015). Research electives, compulsory research projects, short or longitudinal courses, journal clubs, intercalated degree programs, and other approaches are described in the literature ( Coomarasamy and Khan, 2004; Alahdab *et al.*, 2012; Balajic *et al.*, 2012; Lai *et al.*, 2012; Abu-Zaid *et al.*, 2013; Ahsin *et al.*, 2015; Chang and Ramnanan, 2015; Fielding *et al.*, 2015; Knight *et al.*, 2016). Recent evidence indicates that longitudinal, clinically integrated courses are preferable to develop EBM-related behaviours used in actual clinical practice ( Del Mar *et al.*, 2004; Daher and Amin, 2010; Khader *et al.*, 2011; Cheng *et al.*, 2012; Maggio *et al.*, 2016). However, a lack of consensus appears to exist on the best methods to teach and integrate EBM in UME ( Lam *et al.*, 2004; Johnston *et al.*, 2009). A review of systematic reviews suggests that teaching evidence-based health care should also employ a variety of approaches (e.g. lectures, online learning, journal clubs) to improve skills, knowledge and attitudes towards EBM ( Young *et al.*, 2014).

Some literature has been devoted to medical students’ opinions and perceptions of learning about EBM and research in general. Doing research and learning about EBM have been described as ‘boring’, ‘not interesting’ or ‘unstimulating’ by some students ( Burgoyne *et al.*, 2010; Daher *et al.*, 2010; Fielding *et al.*, 2015; Tiwari *et al.*, 2017). Other negative perceptions include conflict between EBM recommendations, patient preferences, and experience-based medicine, and a lack of context in the preclinical years. Students have also reported dissatisfaction with the level of training, mentorship and other factors provided by their educational institutions ( Butt and Khan, 2008; Alahdab *et al.*, 2012; Abu-Zaid *et al.*, 2013; Feigerlova *et al.*, 2016; Maggio *et al.*, 2016).

Positive perceptions exist; students describe EBM/research skills as essential for the duration of their careers ( Nazha *et al.*, 2015). Furthermore, it appears appreciation for and involvement in EBM/research increases throughout medical school, as students approaching residency are better able to appreciate their relevance and impact ( Siemens *et al.*, 2010).

The pre-clerkship curriculum of the University of Toronto (U of T) MD Program encompasses the first two years of medical school, in which Health Science Research (HSR) is a major component (Supplementary File 1). Through online modules, assigned readings, large group sessions and small group tutorials, students learn how to identify, understand and apply research evidence to the care of patients, populations, health systems, and policy. HSR also includes a longitudinal, independent assignment called the practicum exercise, in which students write an original research protocol, followed by its oral presentation to their peers and tutors. While the student is not required to carry out this research, the protocol may serve as a basis for future study, if so desired. Objective student assessments in HSR include quizzes and exams.

From 2015-2017, second-year medical students at U of T participated in the one-year HSR component. In the 2017-2018 academic year, HSR transitioned into a two-year curriculum, covering the first two years of medical school.

To date, this literature review did not reveal any evidence on the perceptions and attitudes of students toward this specific type of UME EBM and research methods intervention.

The objective of this study was to understand the students’ perceptions as to how HSR prepared them to identify, critically appraise and use research (i.e. to be consumers of research) in their clerkships.

## Methods

### Study design and site

This pilot study was an anonymous survey of medical students completing their fourth and final year at the University of Toronto Faculty of Medicine (Class of 2018). It was hosted online ( www.surveymonkey.com) from 16 May 2018 until 30 June 2018. Students were asked their impressions as to the value and utility of HSR in preparing them to identify, critically appraise and use research (i.e. to be consumers of research) in their clerkships.

### Questionnaire

An extensive literature review informed the development of a twelve-item questionnaire, estimated to require 5-10 minutes to complete (Supplementary File 2). The questionnaire was divided into three parts: (i) demographic information (e.g. age, gender); (ii) students’ impression of HSR’s utility in domains including: how to search for medical literature, and analyse and critically appraise research articles, preparation on how to become a consumer of research, and (iii) student opinions of HSR. In addition, students were asked to describe how well they thought HSR prepared them to be a consumer of research in their upcoming postgraduate training.

### Study sample and recruitment

University of Toronto undergraduate medical students from the Class of 2018, who completed HSR during the 2015-2016 academic year were eligible for the study. There were no exclusion criteria.

After U of T Research Ethics Board approval, the Registrar’s Office from the Faculty of Medicine emailed the survey invitation, containing a link to the online survey, to all 262 eligible students. Students were sent reminder emails by the Registrar’s Office at two weeks, four weeks and at five weeks (one week before the end of the survey). This study was carried out in accordance with the Declaration of Helsinki, including but not limited to ensuring privacy, confidentiality and obtaining informed consent.

### Analysis

Basic descriptive statistics of the sample were performed, using means, standard deviations, medians and proportions as appropriate. The response rate was determined by dividing the number of participants by the maximum possible sample size. Free text responses were analysed via descriptive thematic analysis ( Corbin and Strauss, 2008), an iterative process in which themes, or patterns in the data are developed, described and recorded. Results of the five-point Likert scale items were summarised using proportions and medians. Although Likert scale data is technically ordinal in nature, it has been treated as interval-level data, using parametric or non-parametric analyses ( Sullivan and Martino, 2013); however, the use of the median as the measure of central tendency is preferred ( Jamieson, 2004). For simplicity and intuitiveness, (and because this is a non-inferential study), we chose to compare the proportions of “favourable” responses (agree or strongly agree) versus “not favourable responses” (strongly disagree, disagree and neutral). No inferential statistics or subgroup analyses were planned for this study.

## Results/Analysis

**Figure 1.**
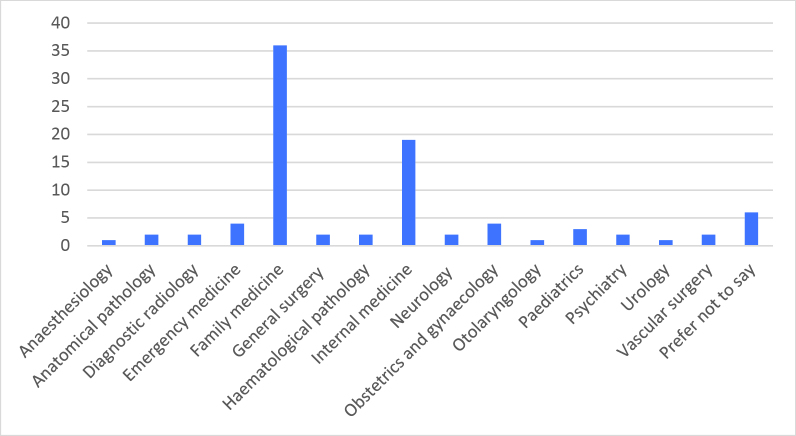
Distribution of residency programs (postgraduate training) to which respondents applied

A total of 26% (67/262) of students participated; their average age was 27 years (SD 2.4, range 24-36). Forty percent were female (27/67). Ninety-six percent of students indicated they had applied to the CaRMS residency (i.e. postgraduate training) match in 2018 (64/67); four percent did not apply (3/67) ( Table 1, Figure 1).

**Table 1.** Demographic information

Gender (n= 67)				
	Male	Female	Prefer not to say	
	39 (58.21%)	27 (40.30%)	1 (1.49%)	
Mean age in years, standard deviation (n=65)				
			27 (2.4)	
Degrees obtained (total respondents=67) ^+^				
Bachelor’s	Master’s	PhD	Non-Canadian MD	Other	58 (86.57%)	18 (26.87%)	6 (8.96%)	0 (0%)	2 (2.99%)
CaRMS match participation 2018 (n=67) *						Yes	No		
	64 (95.52%)	3 (4.48%)		

The Canadian Residency Matching Service (CaRMS) is the organisation that provides application and match services for postgraduate medical training in Canada. *

Participants were able to select more than one answer ^+^

Half of respondents (32/64) either agreed or strongly agreed (i.e. ‘favourable’) that HSR was useful to help them develop a clinically focused search question. Fifty-three percent assigned a rating of agree or strongly agree (34/64) that HSR was helpful in teaching how to search for research to answer specific clinical questions. Only 20% (13/63) of students felt that HSR was useful for learning how to access the grey literature (e.g. blogs, reports, conference proceedings). Approximately half of respondents either agreed or strongly agreed that HSR helped them to critically appraise research articles (50.7%, 32/63), assess study validity (49.2%, 31/63) and assess whether study results could be applied to patient care (50.8%, 32/63). Only 15.9% (10/63) of students agreed or strongly agreed that HSR helped them to disseminate research findings or engage in knowledge translation during clerkship. Responses to the Likert-scale questions on HSR’s utility are reported in Table 2 and 3 and Figures 2 and 3.

**Table 2.** Distribution of responses to the question “The HSR skills on how to search for scientific literature were useful during clerkship for...”

	Strongly disagree	Disagree	Neutral	Agree	Strongly agree	Not applicable/unable to assess	Total	Median	Mean	Standard deviation
Developing a clinically-focused research question	3 (4.69%)	6(9.38%)	23(35.94%)	25(39.06%)	3(4.69%)	4(6.25%)	64	3	3.32	0.90
Developing a clinically-focused search question	3(4.69%)	8(12.50%)	17(26.56%)	28(43.75%)	4(6.25%)	4(6.25%)	64	4	3.37	0.97
Searching for answers to specific clinical questions	3(4.69%)	5(7.81%)	18(28.13%)	30(46.88%)	4(6.25%)	4(6.25%)	64	4	3.45	0.92
Accessing the grey literature (e.g. blogs, research reports, conference proceedings)	6(9.52%)	12(19.05%)	25(39.68%)	12(19.05%)	1(1.59%)	7(11.11%)	63	3	2.82	0.95

Favourable answers (agree or strongly) are shaded in grey.*

**Table 3.** Distribution of responses to the question “HSR skills helped me during clerkship to...”

	Strongly disagree	Disagree	Neutral	Agree	Strongly agree	Not applicable/unable to assess	Total	Median	Mean	Standard deviation
Assess the appropriate use of quantitative research methods	1(1.59%)	8(12.70%)	25(39.68%)	25(39.68%)	1(1.59%)	3(4.76%)	63	3	3.28	0.78
Assess the appropriate use of qualitative research methods	1(1.59%)	10(15.87%)	31(49.21%)	14(22.22%)	1(1.59%)	6(9.52%)	63	3	3.07	0.75
Analyse research findings	1(1.59%)	8(12.70%)	22(34.92%)	28(44.44%)	1(1.59%)	3(4.76%)	63	4	3.33	0.79
Critically appraise research articles	1(1.59%)	8(12.70%)	19(30.16%)	29(46.03%)	3(4.76%)	3(4.76%)	63	4	3.42	0.84
Understand ethical principles in research	2(3.17%)	9(14.29%)	19(30.16%)	27(42.86%)	2(3.17%)	4(6.35%)	63	3	3.31	0.89
Assess the importance of research article results	1(1.59%)	5(7.94%)	25(39.68%)	25(39.68%)	3(4.76%)	4(6.35%)	63	3	3.41	0.78
Assess study validity	1(1.59%)	6(9.52%)	22(34.92%)	27(42.86%)	4(6.35%)	3(4.76%)	63	4	3.45	0.83
Assess whether study results can be applied to patient care	1(1.59%)	7(11.11%)	19(30.16%)	29(46.03%)	3(4.76%)	4(6.35%)	63	4	3.44	0.83
Disseminate research results	6(9.52%)	15(23.81%)	24(38.10%)	8(12.70%)	2(3.17%)	8(12.70%)	63	3	2.73	0.96
Engage in knowledge translation	4(6.35%)	17(26.98%)	22(34.92%)	8(12.70%)	2(3.17%)	10(15.87%)	63	3	2.75	0.93

Favourable answers (agree or strongly) are shaded in grey.*

Figure 2: Distribution of favourable (agree or strongly agree) versus unfavourable (strongly disagree or disagree or neutral) responses to: “The HSR skills on how to search for scientific literature were useful during clerkship for”:

**Figure 2a.**
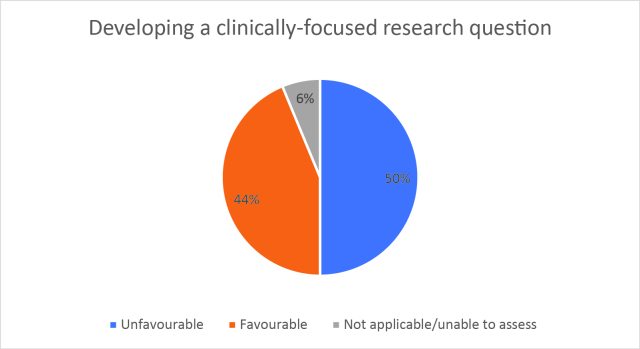
Developing a clinically-focused research question

**Figure 2b.**
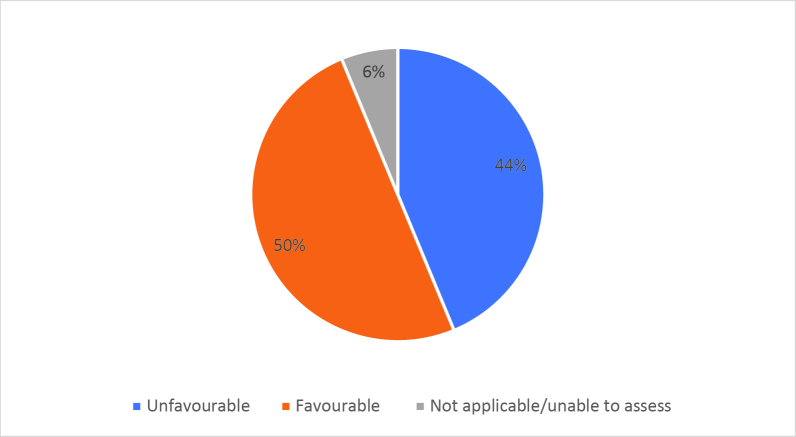
Developing a clinically-focused search question

**Figure 2c.**
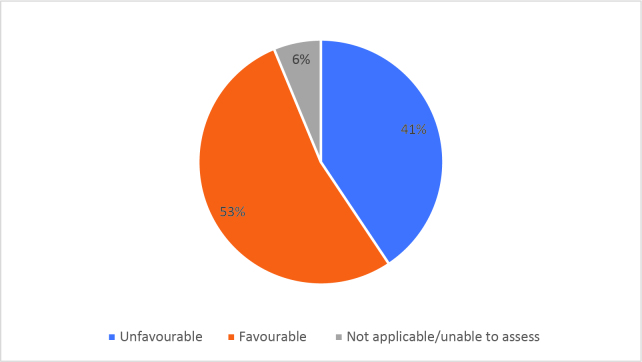
Searching for answers to specific clinical questions

**Figure 2d.**
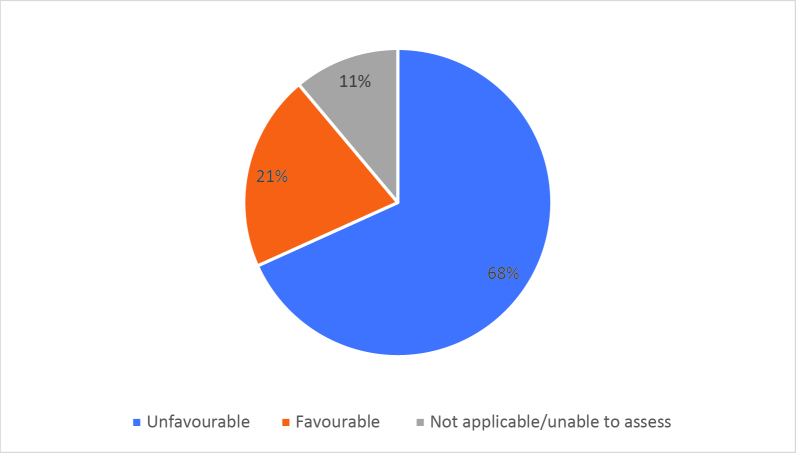
Accessing the grey literature (e.g. blogs, research reports, conference proceedings)

Figure 3: Distribution of favourable (agree or strongly agree) versus unfavourable (strongly disagree or disagree or neutral) responses to: “HSR skills helped me in clerkship to”:

**Figure 3a.**
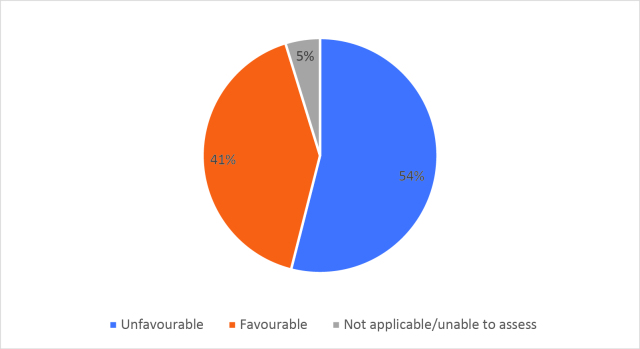
Assess the appropriate use of quantitative research methods

**Figure 3b.**
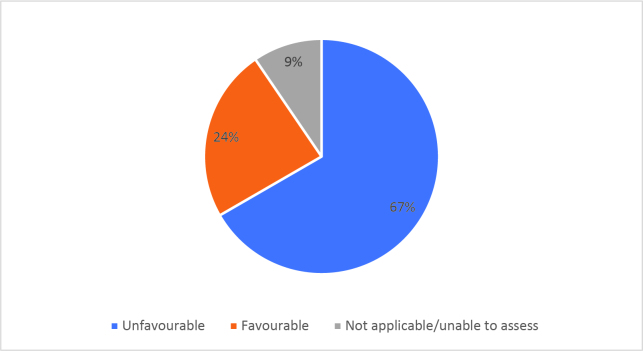
Assess the appropriate use of qualitative research methods

**Figure 3c.**
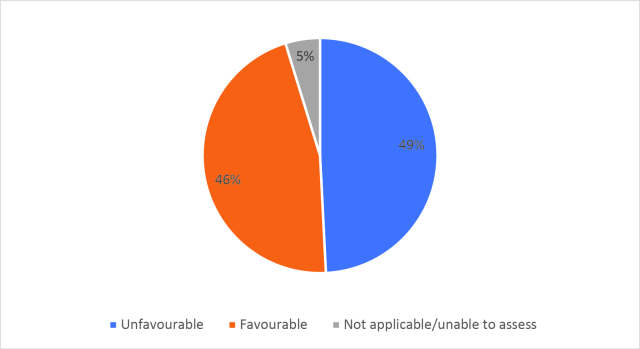
Analyse research findings

**Figure 3d.**
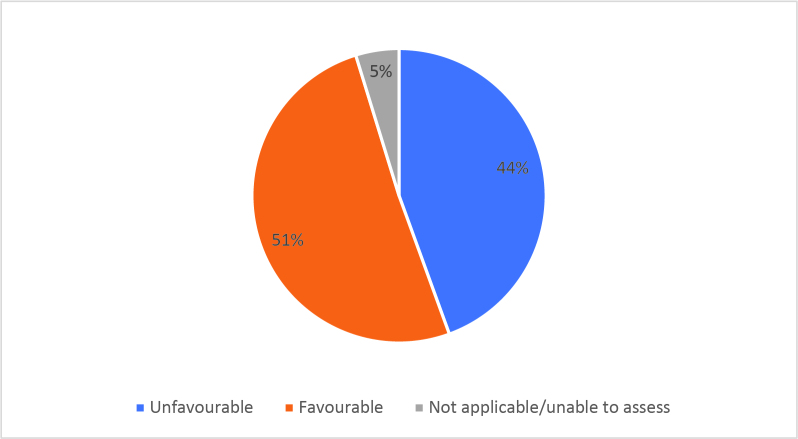
Critically appraise research articles

**Figure 3e.**
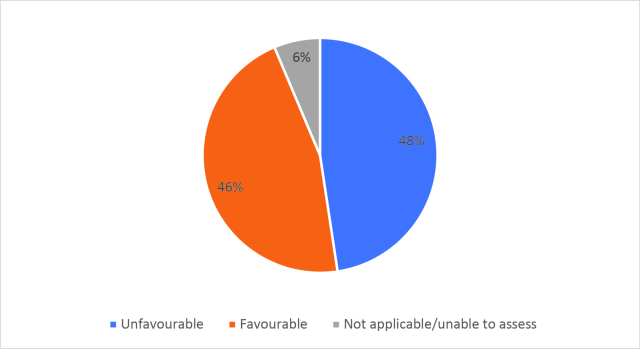
Understand ethical principles in research

**Figure 3f.**
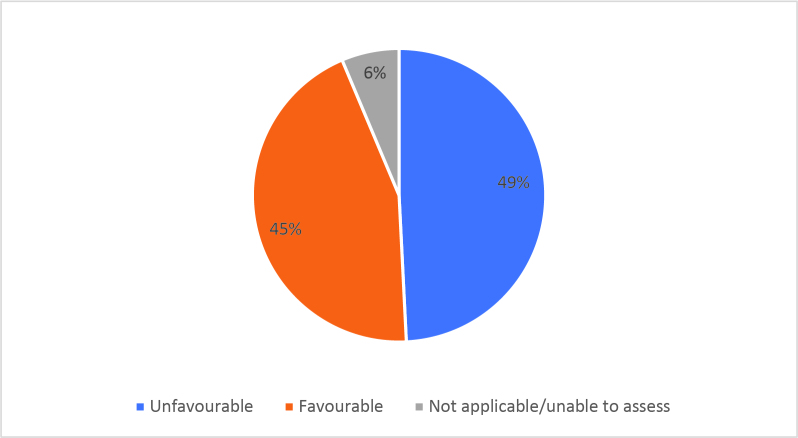
Assess the importance of research article results

**Figure 3g.**
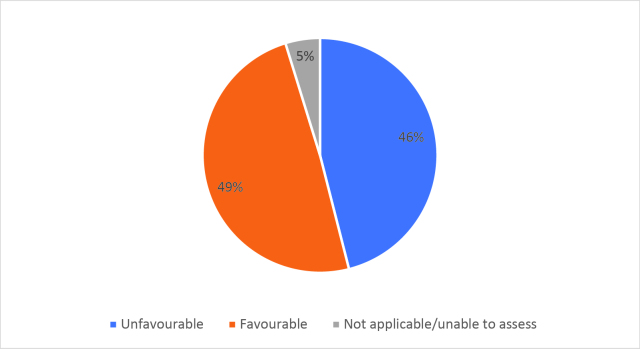
Assess study validity

**Figure 3h.**
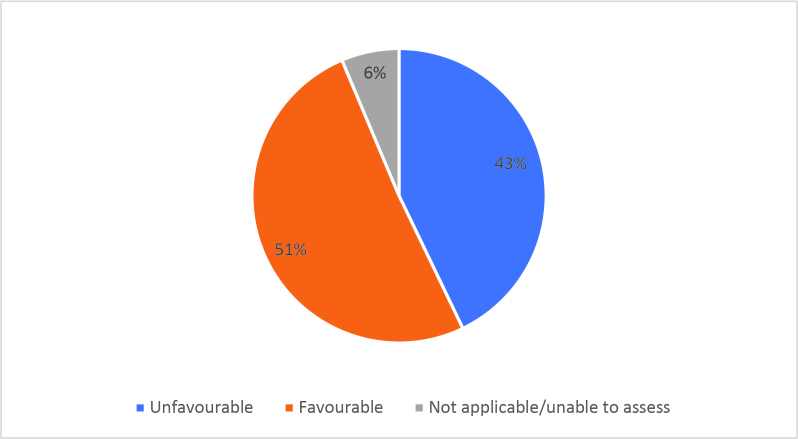
Assess whether results can be applied to patient care

**Figure 3i.**
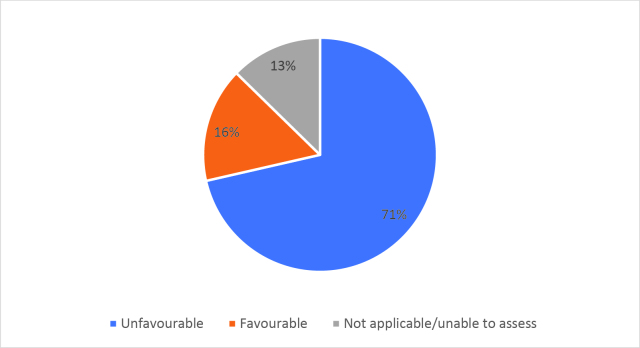
Disseminate research results

**Figure 3j.**
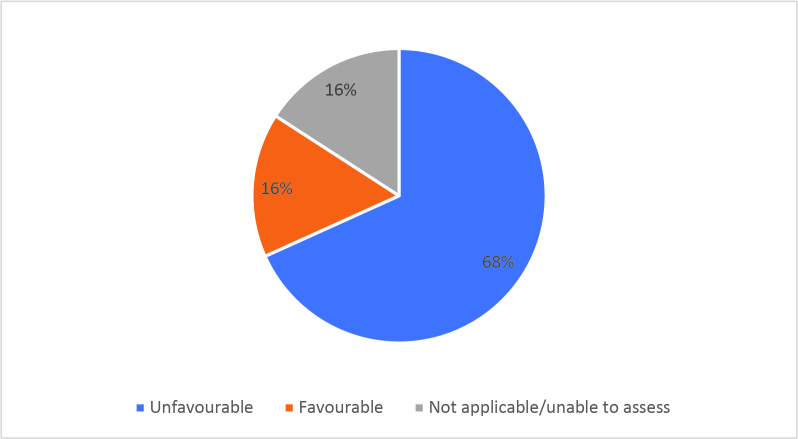
Engage in knowledge translation

Three interesting themes emerged from the free text responses ( Table 4). Most notable was the frequently cited desire for an increased focus on critical appraisal of scientific literature in HSR, to support their preparation to become consumers of research. Some participants also proposed that more practice in searching the literature for specific clinical questions, finding applicable articles, and then critically appraising the articles would be useful, as was participation in mock journal clubs. Students in this sample appeared cognizant of the importance of physicians’ being competent consumers of research, even during clerkship.

**Table 4.** Themes emerging from free-text responses

Desire for greater emphasis on critical appraisal	“[should] be more exercises on critical appraisal and translating clinical situations in to [sic] mini ‘research assignments’”
“...critiquing... research. thats [sic] what we did during clerkship and realistically will be doing during our careers- for the majority of us.”
Less focus on producing hypothetical research greater value in producing real research	“rather than trying to focus on making us primary researchers... not saying this isn’t an important role to be filled by some individuals, but this should not be the focus of a course to a general medical class with developing strong, competent practicing physicians- rather than training researchers”
“more opportunities to be part of research projects”
Greater appreciation for the value of research and research methods during clerkship	“...brought to light the issues with applying research findings to my specific patient/patient population [and] helped me to understand the factors that make evidence strong or weak...”.
“by HSR training us to appraise literature critically, and take their results with a grain of salt”.
“the relevance of course material became more clear once we were exposed to research in clinically-based settings”
“during the course, it was difficult to see the utility of the material... but during clerkship I did appreciate that... HSR helped me understand discussions in journal clubs.”

The second theme was the sentiment that learning to be a producer of research (i.e. writing a protocol as an exercise) was less important than learning to be a consumer of research. Several respondents described physicians all needing to be competent consumers of research as a minimum competency. However, a few suggested adding a requirement to complete an actual research project in HSR or obtaining credit for carrying out extracurricular research in which they were actively involved.

A third theme was students developing a greater appreciation for the importance of learning about research and research methods during clerkship, echoing pre-existing literature. Certain students felt that HSR should have been taught earlier in the curriculum (during the first year of medical school), whereas others suggested an additional review session during clerkship.

Students reported several ways in which HSR helped to prepare them to be consumers of research as clerks, e.g.: learning about different study types and biostatistical concepts, how to ask research questions, and how to find, appraise and use research findings.

During clerkship, some students found HSR to be valuable. Others found it less helpful, for reasons including learning to do critical appraisal in real-world clinical settings during clerkship (as opposed to during HSR), or that HSR was redundant because they had learned the material previously in other courses or degrees. One respondent felt that HSR should be removed from the curriculum.

Participants were asked if their opinions about HSR had changed since completing the course in 2016; 68% (44/64) felt their opinions had not changed. Eleven percent (7/64) felt their opinions had changed; whereas 17% did not know (11/64). Two students declined to answer. Students reported changed opinions because they were able to see the relevance of the material during clerkship. Students whose opinions had not changed felt that the inaugural HSR content and delivery at its inception in the 2015-2016 academic year were not fully developed, or that the material was redundant for students with prior research training. Other students had expected the course to be useful and felt that it had been.

Students were asked to describe, in their own words, how well they thought HSR prepared them to be consumers of research for their upcoming postgraduate training. Answers ranged from “not at all” to “reasonably well”. Two students stated that HSR provided a foundation to develop upon during residency. As mentioned previously, some students felt that non-medical undergraduate courses or postgraduate coursework prepared them better for residency than HSR, or that clinical experiences in clerkship alone had done so.

## Discussion

Although this was a brief, web-based pilot study with a relatively small response rate, it yielded valuable information. Our findings suggest that these students’ perceptions of HSR were variable, and its utility to have a positive impact on research related experiences later in their clinical training was modest.

Most respondents were male (60%). Some literature has discussed gender differences in perceptions of research and EBM amongst medical students ( Abu-Zaid *et al.*, 2013; Feigerlova *et al.*, 2016). For example, male students have reported feeling more competent in research-specific skills compared to females ( Balajic *et al.*, 2012; Zee *et al.*, 2014). It is unknown if a more balanced gender sample would have yielded different results. Other literature has suggested that students interested in surgical specialties tend to be more active in research ( Nikkar-Esfahani *et al.*, 2012), but in our sample, most students had applied to family medicine or internal medicine. We would have surmised that students interested in surgical specialties would have been more represented in our sample, because of a potential increased interest in research. If surgical specialties were less desired in this class, the lower representation of such specialties in our sample might have been proportional. If not, it is unclear why students interested in surgical specialties were less likely to complete the survey than students not interested in surgical specialties.

As previously mentioned, the analysis of Likert scale data can be controversial, but by assessing the proportions of “favourable” responses (agree or strongly agree) versus the “not favourable” we learned that accessing the grey literature, disseminating results and engaging in knowledge translation were the poorest-scoring HSR course objectives. We aim to study if subsequent versions of HSR have better addressed these topics, and if future students have a more favourable opinion these domains, and the course overall, given that the overall ‘favourable’ opinions hovered around the 50% mark.

Some students found HSR very helpful, while others did not. It is noteworthy that as respondents expressed an understanding of the importance of being a consumer of research, the items related to being a consumer of research (e.g. developing clinically-focused search questions or being able to critically appraise research articles) scored relatively higher.

Several students indicated an appreciation for learning about evidence-based medicine and research methods, and the importance of being a consumer of research. It is important to note that these students were still early in their medical training, with limited clinical experience. Several students commented that while most of them would not be producers of research, they would all be consumers of research in some way, and thus the latter should be the focus of HSR.

After gaining more clinical experience during clerkship, some students described an increased appreciation of the content taught in HSR, which agrees with research in this area ( Siemens *et al.*, 2010). Some students also agreed with other research, that HSR should continue to be taught early in medical school ( Holloway *et al.*, 2004; Bradley *et al.*, 2005; Nieman *et al.*, 2009).

Residency program application is a competitive process and demonstrating a proficiency in scholarly activity can increase an applicant’s chances of matching to a desired program ( Burgoyne, O’Flynn and Boylan, 2010; Chang and Ramnanan, 2015). Curiously, students in this sample did not comment on HSR’s utility in helping to secure a residency position, although one respondent did say that HSR helped them begin an actual research project.

Some medical schools require students to complete an actual research project as part of the curriculum ( Chapman *et al.*, 2015; Knight *et al.*, 2016). Interestingly, in our sample, several students mentioned doing an actual project in HSR would have been preferable than the research practicum. While the MD program at U of T no longer requires students to complete an actual scholarly project, scholarly activity is a part of many Canadian postgraduate medical training programs ( Jerzak, Arnold and Haider, 2015). Now that the respondents are residents, it would be interesting to know if they would have a different opinion on the research production component of HSR, analogous to how medical students appear to realise the value of research and research methods teaching after more clinical exposure.

Some students indicated they would have preferred more traditional lectures to learn HSR concepts as opposed to online modules, a return to more structured teaching. Further study will be required to determine the optimal delivery of HSR (e.g. more classroom-based lectures versus predominantly online learning).

### Limitations

Due to time constraints, the questionnaire was not tested with experts beyond the study team or medical students, and thus was not rigorously assessed for content or face validity; neither was it pre-tested. The study period coincided with licensing examinations and graduation, which may have resulted in a smaller sample size than might otherwise have been obtained. The respondents might not be representative of the entire class.

Using Likert scales to capture and summarise opinions also has other challenges in addition to those listed above; for example, one cannot infer that the difference between ‘strongly disagree’ and ‘disagree’ is equivalent to the difference between ‘agree’ and ‘strongly agree’ ( Jamieson, 2004). However, in this purely descriptive study, comparing median results and proportion of favourable to unfavourable responses to different survey items can be helpful to judge relative differences in utility ( Tables 2 and 3, Figures 2 and 3).

It is also unknown if the passage of time (two years) might have influenced students’ recollections. Since the 2015-2016 academic year, HSR has undergone several revisions, most notably into a two-year course, with greater focus on critical appraisal; some of the data in this study may no longer be applicable. As these students have not been followed up with during residency, we do not know if their opinions on HSR have further changed. Furthermore, we were unable to probe responses to further assess why students felt the way they did. Finally, congruent with other research, students’ responses were based upon self-perception; it is unknown whether perceived abilities translated into actual ability (e.g. critical appraisal) ( Caspi *et al.*, 2006; Khan *et al.*, 2001; Alahdab *et al.*, 2012).

## Conclusion

In conclusion, these students from the Class of 2018 from the Faculty of Medicine at the University of Toronto provided a range of perceptions about HSR’s value and utility for identifying, critically appraising and using research in clerkship. HSR’s impact would best be described as modest, based on approximately half of respondents giving a “favourable” rating (agree or strongly agree) in multiple areas. However, aspects of HSR most useful to students included: how to search for research to answer specific clinical questions, how to critically appraise articles, and how to assess their validity and applicability to patient care. Less useful areas included searching in the grey literature and knowledge translation.

Major themes from the data included students’ desire to focus on the consumption of research (especially critical appraisal) as opposed to producing original research, and that an appreciation for EBM and research methods increased over time. The current, two-year version of HSR has already incorporated significantly more critical appraisal, and students’ messages from this study are informing further course development. Future study should address whether students’ educational needs in this area are being better met. Also, strategies should be developed to increase preclinical students’ appreciation for the value of research and research methods, which might increase awareness of their importance during HSR.

Our findings may not be generalisable to the larger U of T medical student population, or medical students in general. However, our observations may be useful to EBM and research methods educators in other centres, particularly as this specific type of educational EBM and research methods intervention is not well represented in existing literature.

## Take Home Messages

• Perceptions of HSR were variable; its utility on research related experiences during clinical training was modest
• Most useful aspects included searching for research, answering specific clinical questions, critical appraisal and assessing articles’ applicability to patient care
• Less useful areas included searching grey literature and knowledge translation
• Major themes were desire for more consumption of research skills (especially critical appraisal, compared to production of research) and the appreciation for EBM and research methods during clerkship
• Future inquiry should assess whether students’ HSR educational needs are being met, and developing strategies to increase preclinical students’ appreciation for research and research methods

## Notes On Contributors

**Dr. Telisha Smith-Gorvie** is a Clinician Teacher and Assistant Professor in the Department of Medicine at the University of Toronto. Her educational focus is health research methodology; she is the course director for the FRCP Emergency Medicine Clinical Epidemiology course at the University of Toronto.

**Dr. Joyce Nyhof-Young** is a qualitative and mixed methods education scientist, medical educator and Curriculum Evaluation Coordinator in the Office of Assessment and Evaluation in the MD Program at the University of Toronto. She is an Associate Professor of Family and Community Medicine.

**Jennifer Ng** graduated from the University of Toronto with a Bachelor of Arts degree with honours in English and Employment Relations. Jennifer has been the Foundations Coordinator for the Health Science Research Component in the MD Program at the University of Toronto since 2015.

**Dr. Tony D’Urzo** is an Associate Professor in the University of Toronto Department of Family and Community Medicine (DFCM), a UTOPIAN researcher in the DFCM, Chair and Co-founder of the Primary care Respiratory Alliance of Canada (PCRC) and former Associate Director of the Health Science Research Component and the Deputy Pre-Clerkship Director, DFCM.

**Dr. Debra Katzman** is a Professor of Pediatrics, in the Department of Pediatrics and University of Toronto (UofT) and Senior Associate Scientist at the Research Institute at the Hospital for Sick Children. Dr. Katzman is the Director for Health Science Research in the MD Program at UofT.

## Appendices

Please see Supplementary Files 1 and 2.

## Declarations

Dr Smith-Gorvie is a co-tutor and is a site lead tutor for Health Science Research. Dr Nyhof-Young was an HSR co-tutor and site lead tutor. Dr Katzman is the HSR Course Director, and Dr D’Urzo was the assistant HSR Course Director at the time of this study. Ms Ng is the coordinator for HSR. The Principal Investigator (Dr Smith-Gorvie) was not involved in HSR during the 2015-2016 academic year.

## References

[ref1] Abu-Zaid A. and Alkattan K. (2013) Integration of scientific research training into undergraduate medical education: a reminder call. Medical Education Online. 18(1), p.22832. 10.3402/meo.v18i0.22832 24149135 PMC3805839

[ref2] Ahsin S. (2015) Reciprocal benefit to senior and junior peers: An outcome of a pilot research workshop at medical university. JPMA. The Journal of the Pakistan Medical Association. 65(8), pp.882–884. 26228337

[ref3] Alahdab F. (2012) Undergraduate medical students’ perceptions, attitudes, and competencies in evidence-based medicine (Ebm), and their understanding of EBM reality in Syria. BMC research notes. 5, p.431. 10.1186/1756-0500-5-431 22882872 PMC3520748

[ref4] Alonso-Coello P. (2009) Perceptions, attitudes and knowledge of evidence-based medicine in primary care in Spain: a study protocol. BMC health services research. 9, p.80. 10.1186/1472-6963-9-80 19445660 PMC2694172

[ref5] Balajić K. (2012) Influence of a vertical subject on research in biomedicine and activities of The Cochrane Collaboration branch on medical students’ knowledge and attitudes toward evidence-based medicine. Croatian Medical Journal. 53(4), pp.367–373. 10.3325/cmj.2012.53.367 22911530 PMC3428824

[ref6] Black M. L. (2013) Summer research training for medical students: impact on research self-efficacy: summer research training for medical students. Clinical and Translational Science. 6(6), pp.487–489. 10.1111/cts.12062 24330695 PMC3868994

[ref7] Bradley P. (2005) Medical students’ and tutors’ experiences of directed and self-directed learning programs in evidence-based medicine: a qualitative evaluation accompanying a randomized controlled trial. Evaluation Review. 29(2), pp.149–177. 10.1177/0193841X04269085 15731510

[ref8] Burgoyne L. N. O’Flynn S. and Boylan G. B. (2010) Undergraduate medical research: the student perspective. Medical Education Online. 15. 10.3402/meo.v15i0.5212 PMC2939395 20844608

[ref9] Butt A. K. and Khan A. A. (2008) Teaching biostatistics and epidemiology in a postgraduate medical institution: are we going in the right direction? Eastern Mediterranean Health Journal = La Revue De Sante De La Mediterranee Orientale = Al-Majallah Al-Sihhiyah Li-Sharq Al-Mutawassit. 14(5), pp.1192–1197. 19161093

[ref10] Caspi O. (2006) Evidence-based medicine: discrepancy between perceived competence and actual performance among graduating medical students. Medical Teacher. 28(4), pp.318–325. 10.1080/01421590600624422 16807169

[ref11] Chang Y. and Ramnanan C. J. (2015) A review of literature on medical students and scholarly research: experiences, attitudes, and outcomes. Academic Medicine: Journal of the Association of American Medical Colleges. 90(8), pp.1162–1173. 10.1097/ACM.0000000000000702 25853690

[ref12] Chapman S. J. (2015) Promoting research and audit at medical school: evaluating the educational impact of participation in a student-led national collaborative study. BMC medical education. 15, p.47. 10.1186/s12909-015-0326-1 25879617 PMC4456723

[ref13] Cheng H. M. (2012) Two strategies to intensify evidence-based medicine education of undergraduate students: a randomised controlled trial. Annals of the Academy of Medicine, Singapore. 41(1), pp.4–11. 22499474

[ref14] Coomarasamy A. and Khan K. S. (2004) What is the evidence that postgraduate teaching in evidence based medicine changes anything? A systematic review. BMJ. 329(7473), p.1017. 10.1136/bmj.329.7473.1017 15514348 PMC524555

[ref15] Corbin J. M. Strauss A. L. and Strauss A. L. (2008) Basics of qualitative research: techniques and procedures for developing grounded theory. 3rd ed. Los Angeles, Calif: Sage Publications, Inc.

[ref16] Critical Appraisal tools (2014) CEBM. Available at: https://www.cebm.net/2014/06/critical-appraisal/( Accessed: 7 February 2020).

[ref17] Daher A. M. and Amin F. (2010) Assessing the perceptions of a biostatistics and epidemiology module: views of Year 2 medical students from a Malaysian university. A cross-sectional survey. BMC medical education. 10, p.34. 10.1186/1472-6920-10-34 20462464 PMC2885405

[ref18] Del Mar C. Glasziou P. and Mayer D. (2004) Teaching evidence based medicine. BMJ (Clinical research ed.). 329(7473), pp.989–990. 10.1136/bmj.329.7473.989 PMC524536 15514319

[ref19] Feigerlova E. (2016) Predictors of high motivation score for performing research initiation fellowship, master 1, research master 2, and phd curricula during medical studies: a strobe-compliant article. Medicine. 95(5), p. e2633. 10.1097/MD.0000000000002633 26844478 PMC4748895

[ref20] Fielding S. (2015) Views of medical students: what, when and how do they want statistics taught? Scottish Medical Journal. 60(4), pp.164–169. 10.1177/0036933015608329 26403572

[ref21] Holloway R. (2004) Teaching and evaluating first and second year medical students’ practice of evidence-based medicine. Medical Education. 38(8), pp.868–878. 10.1111/j.1365-2929.2004.01817.x 15271048

[ref22] Jamieson S. (2004) Likert scales: how to (Ab)use them. Medical Education. 38(12), pp.1217–1218. 10.1111/j.1365-2929.2004.02012.x 15566531

[ref23] Jerzak K. Arnold D. M. and Haider S. (2015) Scholarly success among internal medicine residents in canada. Canadian Journal of General Internal Medicine. 10(3). 10.22374/cjgim.v10i3.54

[ref24] Johnston J. M. Schooling C. M. and Leung G. M. (2009) A randomised-controlled trial of two educational modes for undergraduate evidence-based medicine learning in Asia. BMC medical education. 9, p.63. 10.1186/1472-6920-9-63 19785777 PMC2761870

[ref25] Khader Y. S. Batayha W. and Al-Omari M. (2011) The effect of evidence-based medicine (Ebm) training seminars on the knowledge and attitudes of medical students towards EBM. Journal of Evaluation in Clinical Practice. 17(4), pp.640–643. 10.1111/j.1365-2753.2010.01604.x 21114803

[ref26] Khan K. S. (2001) Assessments in evidence-based medicine workshops: loose connection between perception of knowledge and its objective assessment. Medical Teacher. 23(1), pp.92–94. 10.1080/01421590150214654 11260751

[ref27] Knight S. E. Van Wyk J. M. and Mahomed S. (2016) Teaching research: a programme to develop research capacity in undergraduate medical students at the University of KwaZulu-Natal, South Africa. BMC medical education. 16, p.61. 10.1186/s12909-016-0567-7 26879830 PMC4754994

[ref28] Lai N. M. Teng C. L. and Nalliah S. (2012) Assessing undergraduate competence in evidence based medicine: a preliminary study on the correlation between two objective instruments. Education for Health (Abingdon, England). 25(1), pp.33–39. 10.4103/1357-6283.99204 23787382

[ref29] Lam W. W. T. (2004) Identifying barriers to the adoption of evidence-based medicine practice in clinical clerks: a longitudinal focus group study. Medical Education. 38(9), pp.987–997. 10.1111/j.1365-2929.2004.01909.x 15327681

[ref30] Liabsuetrakul T. (2009) Longitudinal analysis of integrating evidence-based medicine into a medical student curriculum. Family Medicine. 41(8), pp.585–588. 19724944

[ref31] Maggio L. A. (2016) Challenges to learning evidence-based medicine and educational approaches to meet these challenges: a qualitative study of selected ebm curricula in u. S. And canadian medical schools. Academic Medicine: Journal of the Association of American Medical Colleges. 91(1), pp.101–106. 10.1097/ACM.0000000000000814 26200580

[ref32] Nazha B. (2015) Students’ perceptions of peer-organized extra-curricular research course during medical school: a qualitative study. PLoS One. 10(3), p. e0119375. 10.1371/journal.pone.0119375 25764441 PMC4357456

[ref33] Nieman L. Z. Cheng L. and Foxhall L. E. (2009) Teaching first-year medical students to apply evidence-based practices to patient care. Family Medicine. 41(5), pp.332–336. 19418281

[ref34] Nikkar-Esfahani A. Jamjoom A. A. B. and Fitzgerald J. E. F. (2012) Extracurricular participation in research and audit by medical students: opportunities, obstacles, motivation and outcomes. Medical Teacher. 34(5), pp.e317–324. 10.3109/0142159X.2012.670324 22471919

[ref35] Sackett D. L. (1996) Evidence based medicine: what it is and what it isn’t. BMJ. 312(7023), pp.71–72. 10.1136/bmj.312.7023.71 8555924 PMC2349778

[ref36] Siemens D. R. (2010) A survey on the attitudes towards research in medical school. BMC medical education. 10, p.4. 10.1186/1472-6920-10-4 20096112 PMC2823602

[ref37] Srinivasan M. (2002) Early introduction of an evidence-based medicine course to preclinical medical students. Journal of General Internal Medicine. 17(1), pp.58–65. 10.1046/j.1525-1497.2002.10121.x 11903776 PMC1494995

[ref38] Sullivan G. M. and Artino A. R. (2013) Analyzing and interpreting data from likert-type scales. Journal of Graduate Medical Education. 5(4), pp.541–542. 10.4300/JGME-5-4-18 24454995 PMC3886444

[ref39] Tiwari R. Arya R. K. and Bansal M. (2017) Motivating students for project-based learning for application of research methodology skills. International Journal of Applied & Basic Medical Research. 7(Suppl 1), pp.S4–S7. 10.4103/ijabmr.IJABMR_123_17 29344449 PMC5769169

[ref40] Young T. (2014) What are the effects of teaching evidence-based health care (Ebhc)? Overview of systematic reviews. PLoS ONE. Edited by R. S. Phillips. 9(1), p. e86706. 10.1371/journal.pone.0086706 24489771 PMC3904944

[ref41] Zee M. de Boer M. and Jaarsma A. D. C. (2014) Acquiring evidence-based medicine and research skills in the undergraduate medical curriculum: three different didactical formats compared. Perspectives on Medical Education. 3(5), pp.357–370. 10.1007/s40037-014-0143-y 25395228 PMC4235807

