# Supplementary material for: Medical student perceptions of research training on patient care during clerkship
Source: MedEdPublish (2016). 2020 May 21;9:107. [Version 1] doi: 10.15694/mep.2020.000107.1 (PMC10702863; doi:10.15694/mep.2020.000107.1)
Supplement: Peer review history

# Reviewer report: Reviewer response for version 1

**DOI:** 10.21956/mep.19999.r30465

**Published:** 2020-10-14

**Authors:** Ken Masters

**Affiliations:** 1 Sultan Qaboos University

This review has been migrated. The reviewer awarded 3 stars out of 5 An interesting paper on medical student perceptions of research training on patient care during clerkship.The background to the research is well described and grounded in the literature, and indicates that there is a need for the students’ perceptions of the current research course (HSR) at the institution. The study has been well performed, and the results reasonably well presented (See comments below). Although a small study with a low response rate, it does have some value, but the paper also has several issues that need to be addressed.• The methods should indicate the software that was used for both the quantitative statistics and (if any) for the qualitative data theming. • Demographic data were gathered, but then not used (apart from a single mention in the text and then repeated in Table 1). As a rule, no data should be gathered, unless there is a reason and the data are used for that reason. In this case, the data could be used for one or both of:o Comparing the sample demographics to the entire class: this would be especially useful because the response rate is so low.o Testing for any significant differences based on demographics, especially gender (as this is raised as a possibility in trhe Discussion).• From what I can see, the charts (Figure 2a – 3j) mostly replicate the data that are contained in the Tables, although they do create categories. Presentation in so many rather simplistic charts is really not necessary, and lengthens the paper with unnecessary scrolling. I can see that the authors discuss the categories in their Discussion, and so may wish to keep these category data in the body of the paper – in which case, they can be better presented in tables, or perhaps even as a part of the table giving the original data. If the authors really do wish to display the charts, then they should rather place the charts into a supplementary file. • This is a minor, but necessary point: In the results, there is a need to be consistent in percentage rounding and decimal places. At one stage, 32/63 is given as 50.7%, and later as 50.8%. Sometimes, percentages are given to no decimal places, sometimes to one decimal place, and sometimes to two decimal places. (It’s generally fine in the Discussion to round down to whole numbers, but not in the Results).So, a nice read, but there are issues to be addressed. They should be addressed reasonably easily. I look forward to Version 2 of the paper in which these issues are addressed.

Reviewer Expertise:

NA

No decision status is available

# Reviewer report: Reviewer response for version 1

**DOI:** 10.21956/mep.19999.r30464

**Published:** 2020-07-06

**Authors:** Ben Canny

**Affiliations:** 1 Monash University

This review has been migrated. The reviewer awarded 3 stars out of 5 This article examines the perceptions of final year year medical students on the utility for clinical rotations of a course in Health Science Research (HSR). Of note, the course had a significant focus on Evidence Based Medicine (EBM). It is arguable whether the course constitutes research training, as claimed in the title, though it appears to push students beyond many standard courses in EBM. The results suggest that students are somewhat equivocal in their enthusiasm for the value of the training, with approximately equal proportions agreeing, or disagreeing, with the utility of the course. Free text responses suggested that student saw less value in some of the "research specific" aspects of the course, but appreciated or desired greater clinical context for some of the skills. Approximately only 25% of the eligible population completed the survey, and the authors rightly question the generalisability of their findings. It would have been useful to understand how the demographics of the population (age, sex, education) matched the class overall. Also, though I appreciate the numbers may be too small for allow his analysis, it would be of interest to see if observations differed between the various career intentions of the respondents.The article would have strengthened by a description of the learning objectives and assessment methods of the clerkship rotations, as this may help put the students' perspectives in context. Also, though beyond the scope of this article, it would be of interest to reflect on long-term, rather than acute, effects of training, as graduates forge their careers.

Reviewer Expertise:

NA

No decision status is available

# Reviewer report: Reviewer response for version 1

**DOI:** 10.21956/mep.19999.r30463

**Published:** 2020-06-11

**Authors:** Richard Hays

**Affiliations:** 1 James Cook University

This review has been migrated. The reviewer awarded 4 stars out of 5 I am not surprised by the findings of this small study in one medical school, but as an academic clinician i must confess to being a little disappointed. My experience would suggest agreement with the finding that medical students are not necessarily as a group interested in developing research skills. Only a minority will wish to pursue active research careers. However, I wonder if the results were biased by asking only senor students, who (perhaps rightly) are focused on graduating into the clinical workforce. I also wonder if medical schools offer a broad enough perspective and opportunities outside of the 'traditional' health sciences research paradigm? There are many other fields of research and some are important to certain careers. Reflecting on my own experience, my interests in research were more in understanding human behaviour, and I had an opportunity to work on a project with an psychologist. This was very interesting and satisfying. Later on I gained further training in educational psychology and have worked since in a combination of clinical and education practice, although both were after completing specialty training. The role of research in undergraduate programs may be more to 'plant seeds' that develop later, rather than to produce research-active graduates.

Reviewer Expertise:

NA

No decision status is available
